# Potential prognostic factors for delayed healing of common, non‐traumatic skin ulcers: A scoping review

**DOI:** 10.1111/iwj.13100

**Published:** 2019-02-28

**Authors:** David A. Jenkins, Sundus Mohamed, Joanne K. Taylor, Niels Peek, Sabine N. van der Veer

**Affiliations:** ^1^ NIHR Greater Manchester Patient Safety Translational Research Centre The University of Manchester Manchester UK; ^2^ Health e‐Research Centre, Centre for Health Informatics, Division of Informatics, Imaging and Data Science, School of Health Sciences, Manchester Academic Health Science Centre The University of Manchester Manchester UK; ^3^ Manchester University NHS Foundation Trust Manchester UK; ^4^ NIHR Manchester Biomedical Research Centre, Faculty of Biology Medicine and Health The University of Manchester Manchester UK

**Keywords:** diabetic foot ulcers, healing, pressure ulcers, prognostic factors, venous leg ulcers

## Abstract

Healing of non‐traumatic skin ulcers is often suboptimal. Prognostic tools that identify people at high risk of delayed healing within the context of routine ulcer assessments may improve this, but robust evidence on which factors to include is lacking. Therefore, we scoped the literature to identify which potentially prognostic factors may warrant future systematic reviews and meta‐analyses. We conducted electronic searches in MEDLINE and Embase to identify studies in English published between 1997 and 2017 that tested the association between healing of the three most common non‐traumatic skin ulcers encountered by health care professionals (venous leg, diabetic foot, and pressure ulcers) and patient characteristics, ulcer characteristics, and results from clinical investigations. We included 42 studies that investigated factors which may be associated with the healing of venous leg ulcers (n = 17), diabetic foot ulcers (n = 15), and pressure ulcers (n = 10). Across ulcer types, ulcer characteristics were most commonly reported as potential prognostic factors for healing (n = 37), including the size of the ulcer area (n = 29) and ulcer duration at first assessment (n = 16). A total of 35 studies investigated the prognostic value of patient characteristics (n = 35), including age (n = 31), gender (n = 30), diabetes (n = 22), smoking status (n = 15), and history of deep vein thrombosis (DVT) (n = 13). Of these studies, 23 reported results from clinical investigations as potential prognostic factors, with the majority regarding vessel quality. Age, gender, diabetes, smoking status, history of DVT, ulcer area, and ulcer duration at time of first assessment warrant a systematic review and meta‐analysis to quantify their prognostic value for delayed ulcer healing.

## 1 BACKGROUND

Venous leg ulcers and pressure ulcers are the most common types of complex, non‐traumatic skin ulcers, each with an estimated point prevalence of around 0.3% in the United Kingdom and between 0.05% and 1.52% in the United States.^1^ Foot ulcers in people with diabetes are also relatively common, with a total UK point prevalence of 0.1%,^2^ which amounts to a 5.5% prevalence in the UK diabetic population.^3^ In North America, ulcer prevalence in the diabetic population is estimated to be 13%.^3^

Skin ulcers can be exceedingly painful and distressing for patients and can impair independence and health‐related quality of life.^4^ The care of these complex ulcers is costly to health services, largely because of the volume of nursing time required. For example, in 2016, the annual cost for treating and managing pressure ulcers in the United Kingdom and the United States was estimated at approximately £2.6 billion^5^ and $9.1 to $11.6 billion,^6^ respectively; for foot ulcer in people with diabetes, estimates were £650 million^5^, ^7^ and $9 to $13 billion, respectively.^3^

Whilst many venous leg ulcers, pressure ulcers, and foot ulcers will heal, for some people, this will be protracted, with some never fully healing. A recent study of 247 people with venous leg ulcers reported that 62% of ulcers had healed within 24 weeks, with the other 38% remaining unhealed at the time of follow up.^8^ Another study^9^ on venous leg ulcers reported a median time to healing in three treatment arms of 84, 77, and 91 days. Studies in diabetic foot^10^ and pressure ulcers^11^ have reported a median healing time of 10 weeks.

Prognostic tools are used in several disease areas to identify patients at risk of a certain outcome and to aid clinical decisions or manage resources.^12^ There is scope to use such tools to predict slow healing risk in those with common, non‐traumatic skin ulcers. Yet, there is no overarching intelligence about who is likely to heal and who is not, who may benefit from targeted healing‐oriented intervention and who will not, and whether we can use resources more efficiently by targeting those at highest risk.

Prognosis research is invaluable in providing ways to answer these questions. However, available tools only use ulcer characteristics (eg, ulcer size, tissue type) to predict healing time,^13^ whereas others require variables that may be difficult to measure as part of regular ulcer assessments in practice (eg, ankle‐brachial pressure index).^14^ To further improve these tools, we need robust evidence on what factors may have prognostic value for assessing the risk of delayed ulcer healing. Parker et al reviewed the literature up to 2013 in order to identify risk factors for delayed venous leg ulcer healing,^15^ informing the subsequent development of a prognostic tool.^16^ However, they applied a limited set of search terms related to prognostic factors, which was not in line with Cochrane guidance (http://methods.cochrane.org/prognosis). Furthermore, it is unclear to what extent Parker's findings generalise to other ulcer types.

Therefore, we conducted a scoping review of the literature to gain insight into which factors may have potential prognostic value for delayed healing of several common non‐traumatic skin ulcer types, with a focus on factors that can be collected as part of routine ulcer assessments. We expect our findings to guide future decisions about what potential prognostic factors should be prioritised for further investigations through systematic reviews and meta‐analyses.

## 2 METHODS

We designed and reported our scoping review guided by Arksey and O'Malley's^17^ framework, further recommendations by Levac et al^18^ and the Preferred Reporting Items for Systematic Reviews and Meta‐Analyses statement.^19^

### 2.1 Search strategy

We searched Medline and Embase via Ovid for publications in English between 1997 and 2017. The search syntax consisted of terms related to the three ulcer types (informed by input from the Cochrane Wounds group, http://wounds.cochrane.org), combined with terms for prognosis, prognostic factors, and prediction models, as recommended by the Cochrane Prognosis Methods Group (http://methods.cochrane.org/prognosis); the full search syntax is available in Appendix S1.

### 2.2 Study selection

We assessed the eligibility of studies through a two‐stage screening process. Studies were eligible if they adhered to the following inclusion criteria:

- Conducted in an adult population with venous leg ulcers, diabetic foot ulcers, or pressure ulcers. These three non‐traumatic skin ulcers types were selected because of their high prevalence. Other less common ulcer types, such as vasculitic ulcers, were therefore excluded. Traumatic ulcers, such as those arising from surgery or damage to the skin caused by thermal or chemical means, were excluded because of the different aetiology and management of these ulcer types.
- Investigating ulcer healing as an outcome. We accepted authors' definitions of ulcer healing and excluded studies that focused on other outcomes, such as ulcer infection or recurrence.
- Performed a statistical test on the relationship between individual, potential prognostic factors and ulcer healing as an outcome. We included results of clinical investigations as factors (eg, common blood and urine tests, ankle‐brachial pressure index, transcutaneous oxygenation saturation measurements) but excluded those for which measurement would be impractical in the context of routine ulcer assessments (eg, genetic factors). We also excluded papers that did not report significance levels for individual factors when evaluating a score or model combining multiple factors because this would make them ineligible for inclusion in future meta‐analyses.
- Designed as an observational cohort study. We excluded randomised controlled trials (RCTs) because they aim to determine the effect of treatment on ulcer healing while minimising the impact of other influencing factors, whereas these factors are the primary focus of our review. We did include studies that retrospectively used data from a single arm of a trial.
- Original studies, thereby excluding review articles and contributions to conference proceedings.

We first screened the titles and abstracts of all articles. Two authors (D.J. and S.M.) independently screened 50% of articles each and a random sample of 10% in duplicate. Any disagreement was solved through discussion. For all studies deemed relevant, the full text was reviewed using the same screening procedure as in the first stage.

### 2.3 Data extraction and synthesis

We developed a structured form to aid extraction of items related to: general study characteristics (year of publication, country, study setting), study population (type of ulcer, sample size), length of follow up, if data collection was retrospective or prospective, and association between potential prognostic factors and the outcome (outcome definition, factors for which an association with the outcome had been tested [ie, potential prognostic factors], statistical method used). Data for all papers were extracted by one author (D.J.); data from a random sample of 20% of included studies were extracted independently by a second author (S.M.), with discrepancies solved through discussion.

Two researchers (S.V. and J.T., who is a clinician with experience of managing skin ulcers) independently reviewed all factors and categorised them as patient characteristics (eg, age, comorbidities, history of ulcers), ulcer characteristics and treatment (eg, size, depth, duration), or results from clinical investigations (eg, ankle‐brachial pressure index, serum albumin). Ulcer characteristics and treatment were defined as any observation, measurement, or treatment specific to the current ulcer site and immediate surrounding area. Clinical investigations included clinical measurements, clinical imaging, biochemical analysis of blood and urine, and microbiology results. Where needed, the same two researchers independently grouped similar factors into further subcategories to aid the synthesis of results, with discrepancies solved through discussion. For example, the “Patient characteristics” category included subcategories, such as “Socio‐economic status” (consisting of, eg, marital status, income, and educational attainment) and “Cardiovascular disease” (covering conditions such as congestive heart failure, peripheral arterial disease, and angina).

In order to identify potential prognostic factors that may warrant a systematic review, we selected factors that had been investigated by at least 10 studies and, for these factors, assessed if they had been defined in ways that were comparable across studies.

## 3 RESULTS

**Figure 1:**
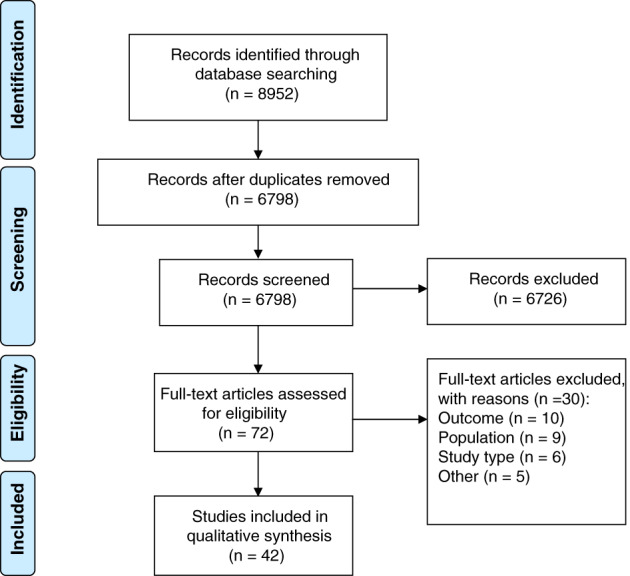
Flow diagram for the identification of relevant studies

The search yielded 6798 unique studies. We reviewed the full text of 72 studies, of which 42 were included. The most common reason for exclusion was that studies did not report ulcer healing as an outcome. Figure 1 shows the flow chart for study selection.

### 3.1 Characteristics of included studies

Table 1 displays the characteristics of included studies. Of the 42 studies included, 16 had been conducted in the United States, and for all three ulcer types, this was the most common country of origin. Most studies took place in an outpatient (26/42) or inpatient (8/42) setting; one study included patients from both.^32^ Studies in community settings (2/42) and nursing homes (2/42) were less common.

**Table 1:** Characteristics of included studies

Reference	Publication year	Country	Setting	Wound type	Sample size	Maximum follow up	Data collection	Outcome	Statistical methods^a^
Abbade et al^20^	2011	Brazil	Outpatient	VLU	90	>10 years	NR	Ulcer not healed after >10 years	*t* test and regression
Barwell et al^21^	2000	United Kingdom	Community	VLU	587	24 wk	Prospective	Time to healing	Cox regression
Beckert et al^22^	2006	Germany	Outpatient	DFU	1000	1 year	Prospective	Time to healing	Cox regression
Berlowitz et al^23^	1998	United States	Nursing homes	PU	819	6 mo	Retrospective	Healed at follow‐up	*t* test and regression
Cardinal et al^24^	2009	United States	NR	VLU	338	12 wk	Retrospective	100% closure	Regression
Chaby et al^25^	2013	France	Outpatient	VLU	104	24 wk	Prospective	Healed at follow‐up	Regression
Christman et al^26^	2011	United States	Outpatient	DFU	183	“Patient‐specific”	Retrospective	Wound area change per day	Regression
Gohel et al^27^	2005	United Kingdom	Outpatient	VLU	1186	24 wk	Prospective	Healed at follow‐up	Cox regression
Hjerppe et al^28^	2010	Finland	Outpatient	VLU	50	12 wk	Prospective	Healed at follow‐up	*χ* ^2^ tests
Horn et al^29^	2015	United States	Outpatient	PU	NR^b^	NR	Retrospective	Healed at follow‐up	*t* test and regression
Ince et al^30^	2007	United Kingdom	Outpatient	DFU	449	1 year	Prospective	Time to healing	Cox regression
Jemec^31^	1999	Denmark	Outpatient clinic	VLU	79	18 mo^c^	Prospective	Wound size and healed	*χ* ^2^ tests and regression
Jones and Fennie^32^	2007	United States	Inpatient and outpatient	PU	114	6 mo	Retrospective	Healed at follow up	*t* test
Kantor and Margolis^33^	1999	United States	NR	VLU	104	24 wk	Retrospective	Healed at follow up	Wilcoxon rank sum
Kapoor et al^34^	2008	United States	Nursing homes	PU	2666	90 d	Retrospective	Healed at follow up	Regression
Labropoulos et al^35^	2011	United States	NR	VLU	127	6 mo	Prospective	Not‐healed by 6 mo	*t* test
Margolis et al^36^	2003	United States	Outpatient	DFU	19 280	20 wk	Retrospective	Healed at follow up	Regression
Margolis et al^37^	1999	United States	Outpatient	VLU	260	24 wk	Retrospective	Healed at follow up	Regression
McGinnis et al^38^	2013	United Kingdom	Outpatient	PU	140	2 years	Prospective	Time to healing	Cox regression
Meaume et al^39^	2005	France	Outpatient	VLU	330	6 wk	Prospective	Area reduction of >40%	Regression
Moffatt et al^40^	2009	United Kingdom	Inpatient and outpatient	VLU	113	48 wk	Prospective	Time to healing	Cox regression
Monami et al^41^	2008	Italy	Inpatient	DFU	80	6 mo	Prospective	Healed	*t* test and cox regression
Oyibo et al^42^	2000	United Kingdom and United States	Outpatient	DFU	194	18 mo	Prospective	Area and time to healed	Correlation and cox regression
Park^43^	2014	Korea	Inpatient critical care	PU	155	1 year	Retrospective	Healed	*t* test and regression
Parker et al^44^	2016	Australia	Outpatient	VLU	247	24 wk	Retrospective	Healed at follow up	*t* test and regression
Rhou et al^45^	2015	Australia	Outpatient	DFU	107	12 wk	Retrospective	Healing rate and healed at follow up	*t* test and regression
Ribu et al^46^	2008	Oslo, Norway	Outpatient	DFU	99	12 mo	Prospective	Healed at follow up	*t* test and anova
Scotton et al^47^	2014	Finland	Outpatient	VLU	94	>1 year	Retrospective	>50% reduction at 6 and 12 mo	Regression
Snyder et al^48^	2010	United States	Outpatient	DFU	250	12 wk	Retrospective	Wound closure	*t* test
Sung and Park^49^	2011	Korea	Inpatient critical care	PU	158	NR	Retrospective	PUSH scale healing^d^	*t* test and regression
Takahashi et al^50^	2009	United States	Wound service in primary care	PU	440	6 mo	Retrospective	Healed at follow up	Regression
Taylor et al^51^	2002	United Kingdom	Outpatient	VLU	325	104 wk	Retrospective	Time to healed	Cox regression
Vedhara et al^52^	2010	United Kingdom	Outpatient	DFU	93	24 wk	Prospective	Healed at follow up	Regression
Wallenstein and Brem^53^	2004	United States	Inpatient	PU	45	12 wk	Prospective	Wound size, time until closure, and % reduction	Gompertz model^e^
Wang et al^54^	2014	China	Inpatient	DFU	194	1 year	Retrospective	Healed at follow up	*t* test and regression
Warriner et al^55^	2011	United States	NR	DFU	120	12 wk	Retrospective	Healed at follow up	Fisher test
Wielen et al^56^	2016	Switzerland	Inpatient	PU	55	Hospital length of stay	Prospective	Time until closure	*t* test
Wipke‐Tevis and Stotts^19^	1998	United States	Inpatient	VLU	25	4 wk	Prospective	Wound‐healing rate	Correlation and contingency
Yang et al^57^	2016	Canada	Outpatient	VLU	522	1 year	Retrospective	Healed at follow up	Regression
Yotsu et al^58^	2014	Japan	Inpatient	DFU	84	NR	Prospective	Healed	*t* test and anova
Zimny and Pfohl^59^	2005	Germany	Outpatient	DFU	41	>10 wk	Prospective	Healing time and wound area	Correlation
Zimny et al^10^	2002	Germany	Outpatient	DFU	31	25 wk	Prospective	Healing time and wound area reduction	Correlation and regression

Abbreviations: anova, analysis of variance; DFU, diabetic foot ulcer; NR, not reported; PU, pressure ulcer; PUSH, pressure ulcer score for healing; VLU, venous leg ulcer.

a Statistical method/test performed to determine if factors were associated with healing.

b Patient numbers not reported, but paper reported 7349 body pressure ulcers and 2112 heel pressure ulcers.

c Mean follow up.

d PUSH which uses a score from 0 to 10 based on ulcer characteristics.

e Univariate time series model considering the relationship between an outcome and time (eg, area‐time relationship).

Of the three ulcer types, venous leg ulcers were considered in 17 of 42 of the studies, foot ulcers in 15 of 42, and pressure ulcers in 10 of 42. Sample sizes (ie, participants recruited) ranged from 25^19^ to 19 280,^36^ with a median of 155 across studies. The majority of studies included fewer than 1000 patients (38/42). The maximum follow‐up period was reported in 35 studies and ranged from 4 weeks^19^ to greater than 10 years,^20^ with a median follow up of 24 weeks. Most studies (38/42) had a follow up of a year or less, with only one study^20^ reporting a follow up longer than 2 years. In two studies, follow up depended on length of hospital stay^26^ or time to the next clinic visit.^49^

Most studies (24/42) defined ulcer healing as a dichotomous outcome indicating whether the ulcer had healed (yes/no); another 10 used time to complete healing. Other definitions were: percentage of ulcer area reduction^39^, ^47^; healing rate^19^; and the pressure ulcer scale for healing score,^49^ which comprises of sub‐scores for ulcer size, tissue type, and exudate. Four studies considered more than one definition.^10^, ^45^, ^53^, ^59^ Overall, almost half of the studies (20/42) were prospective, but only three investigated pressure ulcer healing.^38^, ^53^, ^56^ The most common statistical method used to analyse the data was regression analysis (30/42), and this was Cox regression in nine cases. *t* tests were the second most used statistical analysis performed and was used in 15 studies, some of which also used regression.

### 3.2 Potential prognostic factors

Table 2 shows the result of the categorisation of potential prognostic factors for which studies investigated the association with ulcer healing; see Appendix S2 for the factors as originally reported by the authors of the studies. The number of factors investigated in individual studies ranged from 2 to 45 (median, 15). Factors regarded patient characteristics, ulcer characteristics, or results of clinical investigation in 35 of 42, 37 of 42 and 23 of 42 studies, respectively. Six studies investigated the prognostic value of factors related to only one category, whereas 14 investigated an association with ulcer healing for factors pertaining to two categories, and 22 investigated potential factors related to all three categories.

**Table 2:** Factors investigated in each included study as potential prognostic factors for ulcer healing; [number] reflects the number of individual factors assigned to that subcategory in case there was more than 1

Reference	Total no. of factors investigated	Potential prognostic factors		
		Patient	Ulcer	Clinical investigation
Diabetic foot ulcers (15 studies)				
Beckert et al^22^	4	Local physical manifestations of poor vessel quality	Depth, location, no. of ulcers	
Christman et al^26^	15	Age, blood pressure [2], BMI, body temperature, cardiovascular disease, ethnicity, gender, neuropathy, pulse, smoking status	No. of ulcers	Glycaemic control, inflammatory markers, lipid profile
Ince et al^30^	12	Age, cardiovascular disease, diabetes [2], gender, non ulcer infection, socio‐economic status [2]	Area, depth, location, time to assessment	
Margolis et al^36^	7	Age, gender	Area, duration, no. of ulcers, response to treatment, severity	
Monami et al^41^	8	Depression, other comorbidities and health issues, smoking status	Area, duration, severity	Glycaemic control, quality of vessels
Oyibo et al^42^	9	Age, diabetes [2], gender	Area, depth, location, ulcer infection	Quality of vessels
Rhou et al^45^	26	Age, alcohol consumption, antibiotics use, cardiovascular disease [2], diabetes, gender, kidney disease, medications as proxy for comorbidities/other health issues [5], smoking status	Area, depth, ulcer infection	Alkaline phosphatase, glycaemic control, liver profile [4], renal function, serum albumin/total protein, urate
Ribu et al^46^	10	Cognitive status and mental health, Cognitive status and mental health [2], Functional status [3], Overall health [3], Pain		
Snyder et al^48^	2		Area, response to treatment	
Vedhara et al^52^	21	Age, BMI, cancer, cardiovascular disease [2], cognitive status and mental health, depression, diabetes, gender, history of previous ulcers, hypertension, musculoskeletal disease, neuropathy, other comorbidities and health issues, smoking status, socio‐economic status [2]	Area, ulcer infection	Glycaemic control, quality of vessels (imaging)
Wang et al^54^	32	Age, blood pressure [2], cardiovascular disease, diabetes [3], gender, history of previous ulcers, neuropathy, smoking status, socio‐economic status	Area, duration, location, severity	Glycaemic control [2], inflammatory markers [2], lipid profile, positive microbiology, quality of vessels [2], renal function [3], serum albumin/total protein [2], serum haemoglobin, thyroid function, urine albumin
Warriner et al^55^	3		Area [3]	
Yotsu et al^58^	19	Age, diabetes, gender, history of previous ulcers, kidney disease, other comorbidities and health issues	Area [2], location, necrosis, no. of ulcers, severity, ulcer infection	Quality of vessels [3], renal function, serum albumin/total protein, serum haemoglobin
Zimny and Pfohl^59^	2		Area [2]	
Zimny et al^10^	2		Area [2]	
Pressure ulcers (10 studies)				
Berlowitz et al^23^	19	Age, functional status [2], gender, hospital admission, immobility [5], incontinence, kidney disease, medications as proxy for comorbidities/other health issues, non ulcer infection, other comorbidities and health issues, socio‐economic status, terminal illness	Ulcer type/aetiology	
Horn et al^29^	45	Age, autoimmune disease [3], BMI, Braden score, cardiovascular disease [2], cognitive status and mental health, diabetes [2], history of previous ulcers, gender, hospital admission, immobility [3], incontinence, kidney disease [4], liver dysfunction, medications as proxy for comorbidities/other health issues [2], medications negatively affecting ulcer healing, musculoskeletal disease, non‐traumatic amputation, nutrition [2], other comorbidities and health issues [4], smoking status, socio‐economic status, transplantation, transplantation	Area, duration [2], location, severity, ulcer infection	Quality of vessels (imaging)
Jones and Fennie^32^	41	Age, BMI, cancer, cardiovascular disease [2], cognitive status and mental health, depression, diabetes, DVT, electrolyte imbalance, ethnicity, gender, history of previous ulcers, hypertension, insurance, Kidney disease, musculoskeletal disease, neuropathy [2], nutrition, other comorbidities and health issues [6], smoking status, socio‐economic status [2], stroke, total number of comorbidities	Area[2], depth, exudate [2], location, necrosis, no. of ulcers [2], severity [2]	
Kapoor et al^34^	11	Age, gender, history of previous ulcers, immobility [3], incontinence [2], terminal illness	Severity [2]	
McGinnis et al^38^	17	Age, Braden score, cardiovascular disease, ethnicity, gender, hospital admission, medications as proxy for comorbidities/other health issues, neuropathy, nutrition, other comorbidities and health issues, pain, smoking status	Area, duration, severity, surrounding skin condition, tissue type	
Park^43^	30	Age, blood pressure, Braden score, cancer, cardiovascular disease [2], diabetes, gender, history of previous ulcers, hypertension, immobility, incontinence, medications as proxy for comorbidities/other health issues, medications negatively affecting ulcer healing, musculoskeletal disease, nutrition [2], other comorbidities and health issues [4], smoking status, stool form	Area, exudate, location, tissue type, ulcer infection	Serum albumin/total protein, serum haemoglobin
Sung and Park^49^	30	Age, blood pressure, Braden score, cancer, cardiovascular disease, cardiovascular disease, diabetes, gender, history of previous ulcers, hypertension, immobility, incontinence, medications as proxy for comorbidities/other health issues, medications negatively affecting ulcer healing, musculoskeletal disease, nutrition, nutrition, other comorbidities and health issues [4], smoking status, stool form	Area, exudate, location, tissue type, ulcer infection	Serum haemoglobin, serum albumin/total Protein
Takahashi et al^50^	23	Age, BMI, cancer, cardiovascular disease [3], diabetes, gender, kidney disease, musculoskeletal disease [2], neuropathy, other comorbidities and health issues [4], stroke	Area, no. of ulcers	Glycaemic control, inflammatory markers, quality of vessels, renal function
Wallenstein and Brem^53^	1		Area	
Wielen et al^56^	10	Age, gender, hospital admission, trauma	Duration, location, severity [2], ulcer status, ulcer type/aetiology	
Venous leg ulcers (17 studies)				
Abbade et al^20^	19	Age, BMI, diabetes, DVT, gender, history of previous ulcers, hypertension, local physical manifestations of poor vessel quality [2], multiparity	Area	Quality of vessels [8]
Barwell et al^21^	12	Age, diabetes, gender, immobility, musculoskeletal disease	Area, duration	Quality of vessels [5]
Chaby et al^25^	41	Age, BMI, cardiovascular disease [2], cognitive status and mental health [3], depression, DVT, functional status [2], gender, history of previous ulcers, insurance [2], kidney disease, local physical manifestations of poor vessel quality [2], musculoskeletal disease [3], other comorbidities and health issues, pain, prior venous surgery, socio‐economic status [8]	Area [2], duration, response to treatment	Quality of vessels [3], serum albumin/total protein, serum haemoglobin
Cardinal et al^24^	33	Age, alcohol consumption, BMI [3], cardiovascular disease [2], diabetes, DVT, gender, history of previous ulcers, hypertension, immobility, local physical manifestations of poor vessel quality [4], musculoskeletal disease [2], other comorbidities and health issues, pain, smoking status, stroke	Area [2], duration, exudate, location, necrosis, no. of ulcers, oedema, response to treatment	Quality of vessels
Gohel et al^27^	12	Age, diabetes, DVT, gender, musculoskeletal disease	Duration, location	Quality of vessels [5]
Hjerppe et al^28^	14	Age, BMI, diabetes, gender, immobility, medications as proxy for comorbidities/other health issues, smoking status		Quality of vessels [7]
Jemec^31^	13	Age	Area, duration	Alkaline phosphatase, electrolytes [2], glycaemic control, inflammatory markers, liver profile, quality of vessels, renal function, serum albumin/total protein, serum haemoglobin
Kantor and Margolis^33^	5		Area [5]	
Labropoulos et al^35^	6	Age, BMI, DVT [3]	Area	
Margolis et al^37^	26	Age, cardiovascular disease [2], diabetes, DVT, ethnicity, gender, hypertension, immobility, insurance, local physical manifestations of poor vessel quality [3], musculoskeletal disease, oedema, other comorbidities and health issues, stroke	Area, duration, no. of ulcers, tissue type [2], ulcer type/aetiology	Quality of vessels [3]
Meaume et al^39^	16	Age, BMI, cardiovascular disease [2], diabetes, DVT, gender, hypertension, musculoskeletal disease, smoking status, vascular surgery	Area, duration, infection, no. of ulcers, recurrent ulcer	
Moffatt et al^40^	23	Age, diabetes, DVT, gender, immobility, local physical manifestations of poor vessel quality [4], musculoskeletal disease [2]	Area, duration, ulcer type/aetiology	Positive microbiology [6], quality of vessels [3]
Parker et al^44^	25	Autoimmune disease, depression, DVT, functional status [2], local physical manifestations of poor vessel quality, medications as proxy for comorbidities/other health issues [3], musculoskeletal disease [3], other comorbidities and health issues, pain, socio‐economic status	Area [2], duration, exudate, oedema, pain, pain, PUSH score, response to treatment, tissue type	
Scotton et al^47^	16	Age, antibiotics use [2], diabetes, gender, hypertension, immobility	Area, duration, location, response to treatment [2], ulcer infection [2], ulcer type/aetiology	Quality of vessels
Taylor et al^51^	31	Age, blood pressure [2], BMI, cardiovascular disease, diabetes, DVT, gender, history of previous ulcers, immobility, local physical manifestations of poor vessel quality, musculoskeletal disease, smoking status, socio‐economic status	Area, duration [2], exudate, location, pain, surrounding skin condition [4], tissue type [4], ulcer status	Quality of vessels [2]
Wipke‐Tevis and Stotts^19^	8	Gender, nutrition [3]		Inflammatory markers, quality of vessels [2], serum albumin/total protein
Yang et al^57^	15	Age [2], BMI, cardiovascular disease [3], diabetes, DVT, gender, hypertension, musculoskeletal disease, smoking status, trauma, vascular surgery	Area	

Abbreviations: BMI, body mass index; DVT, deep vein thrombosis; PUSH, pressure ulcer score for healing.

Empty cells mean that no potential factors were identified.

Medications negatively affecting ulcer healing include steroids, immunomodulating drugs, and anti‐coagulants known to prolong bleeding time.

Local physical manifestations of poor vessel quality include clinical findings such as lipodermatosclerosis, varicose eczema, hyperpigmentation, etc.

PUSH score which uses ulcer characteristics to provide a score between 0 and 10.

#### 3.2.1 Patient characteristics

In the 36 studies investigating patient characteristics, age was investigated as a possible prognostic factor in 31 studies (11 in diabetic foot ulcers, 14 in venous leg ulcers, and 9 in pressure ulcers). This was the most commonly investigated factor, followed by gender, which was considered in 30 studies. Most studies considered age a continuous variable, whereas one used categories.^20^ Socio‐economic status (eg, marital status, educational level, home ownership) was considered in 10 studies. The three comorbidities most commonly investigated as a potential prognostic factor were diabetes (23/42: 7 diabetic foot, 10 venous leg, and 5 pressure ulcer studies); cardiovascular disease (17/42); and musculoskeletal disease (16/42). The subcategory of diabetes was defined homogeneously as most studies discussed diabetes as a dichotomous variable, labelling patients as diabetic or not. However, cardiovascular disease contained angina (n = 2), peripheral arterial disease (n = 6), and several other conditions grouped under this subcategory. Musculoskeletal disease was also very heterogeneous, with rheumatoid arthritis (n = 6) being the most common factor in this subcategory. Other patient characteristics investigated as possible prognostic factors included: smoking status (15/42), history of deep vein thrombosis (DVT; 13/42), body mass index (BMI; 13/42), and immobility (12/42). While smoking status and history of DVT were defined relatively homogeneously across studies (mostly as current smoker yes/no and prior DVT yes/no), immobility contained many definitions, from walking aid use to paralysis; BMI was considered either a continuous or categorical variable with different categories across studies.

#### 3.2.2 Ulcer characteristics

Ulcer characteristics were the most commonly investigated potential prognostic factor (39/42). Overall, 14, 15, and 10 studies investigated the association of at least one ulcer characteristic with the healing of diabetic foot ulcers, venous leg ulcers, and pressure ulcers, respectively. Across ulcer types, the size of the ulcer area was most frequently reported (33/42). Most studies (26/42) defined ulcer areas as the complete area of the ulcer in centimetres squared at baseline (ie, time of the first assessment), with only some studies using a different definition (eg, 90% area reduction in area at 4 weeks^48^ or area change in first 2 weeks^33^). Duration of ulcer at baseline (mostly reported in days and used as a continuous variable in the models) was the next most reported factor (17/42 studies), followed by ulcer location (14/42). However, ulcer location had a number of definitions, and no single definition was used across 10 or more studies; for example, 1 study^29^ considered which side of the body the ulcer was, while others^22^ used the categories of toe ulcer or foot ulcer.

#### 3.2.3 Results of clinical investigations

A total of 23 studies considered the results of clinical investigations a possible prognostic factor for ulcer healing, with 13 of these concerned the assessments of vessel quality (eg, through imaging tests). However, this was very homogeneous and included a variety of investigations for vessel quality (eg, transcutaneous oxygen pressure, skin perfusion pressure, and ankle‐brachial index). Abbade et al^20^ considered results from seven imaging tests possible prognostic factors. Other results from clinical investigations included serum albumin (8/42), serum haemoglobin (7/42), glycaemic control (eg, HAb1C) (7/42), and renal function (eg, estimated glomerular filtration rate) (5/42). Only two studies investigated microbiology clinical investigations.^40^, ^54^

## 4 DISCUSSION

### 4.1 Summary of findings

We conducted a scoping review and identified a body of literature investigating potential prognostic factors for healing of venous leg ulcers, diabetic foot ulcers, and pressure ulcers that could be assessed in routine care settings. We included 42 papers, from which we identified age, gender, smoking status, diabetes, musculoskeletal disease, cardiovascular disease, immobility, ulcer area, ulcer location, ulcer duration at time of first assessment, and vessel quality as the factors that were most often investigated as a potential prognostic factor for ulcer healing. Of these, we selected those that were defined sufficiently homogeneously across studies in order to be investigated in a future systematic review and meta‐analysis; all selected factors are listed under “Recommendations for future research” below.

### 4.2 Relation to other studies

Parker et al^15^ conducted a literature review in 2015 on risk factors for delayed healing in venous leg ulcers, including 27 papers published between 2000 and 2013. Our review updates their work while extending search terms for prognostic factors in line with Cochrane guidance (http://handbook-5-1.cochrane.org/) and broadening the scope to include two additional prevalent ulcer types: diabetic foot and pressure ulcers. There were several studies in Parker's review that were captured in our search but did not meet our inclusion criteria. The main reasons for excluding these papers were: ineligible population (ie, also including people with arterial ulcers), ineligible study design (randomised control trial), ineligible publication type (literature review), or analysing effectiveness of an intervention. Parker's review identified ulcer area, ulcer duration, DVT, and history of previous ulcers as consistently reported risk factors for healing. The former three factors were also identified by our review, which suggests that they might have prognostic value across ulcer types.

### 4.3 Recommendations for future research

Age, gender, diabetes, smoking status, history of DVT, ulcer area, and ulcer duration at time of first assessment were investigated in a substantial number of studies, with definitions being sufficiently homogeneous. These potential prognostic factors therefore each warrant a dedicated systematic review and meta‐analysis. Effect sizes of each factor may vary between ulcer types; applying meta‐regression^60^ would adjust for this. Another option would be to address each ulcer type in a separate meta‐analysis, but this would reduce statistical power. However, there is probably sufficient similarity between ulcer types to warrant a meta‐analysis that pools the results across types, thus increasing statistical power. Individual patient data meta‐analyses^61^ are recommended as the gold standard for reducing heterogeneity and standardisation of definitions, but this will require access to individual patient data of all included studies, which is known to be difficult. Access to individual patient data would also allow access to raw data and not rely on categories used in the studies, which would open up possibilities to investigate additional factors in a meta‐analysis, such as BMI.

Many studies included in our review had short follow‐up times and small sample sizes, which will negatively affect the precision of the individual study effect sizes for the potential prognostic factors. Future meta‐analyses will increase this precision by combining evidence across studies into a single combined estimate of prognostic effect size, which will contribute to a better understanding of prognostic factors for ulcer healing. However, the robustness of findings from meta‐analyses investigating prognostic factors depends on the quality and risk bias of included studies,^17^ and future systematic reviews and meta‐analyses would need to assess this, for example, using the Quality in Prognostic studies (QUIPS) tool.^62^

Results from clinical investigations may have prognostic value for ulcer healing, but the current evidence in observational studies is too scarce to warrant systematic reviews in this area. Therefore, future research may focus on conducting larger cohort studies investigating the association between results from clinical investigation and ulcer healing. While we excluded comparative effectiveness studies, a recent Cochrane review^63^ included RCTs to investigate the prognostic value of protease for the healing of venous leg ulcers, resulting in the inclusion of 11 studies for meta‐analysis. A hierarchical approach or down‐weighting of observational data through the use of a power prior would be advised^64^ in this circumstance.

### 4.4 Strengths and limitations of this review

To our knowledge, this scoping review is the first to investigate potential prognostic factors across the most common ulcer types. We used rigorous and transparent methods, including a reproducible search strategy; duplicate assessment of articles for relevance independently by two authors, and use of a pre‐determined data extraction template. A recent study^65^ assessing the methodological quality of 494 scoping reviews found that only 22% reported a search strategy, 36% had used two reviewers, and 43% had a data extraction template.

Our scoping review also has limitations. We did not consult stakeholders as the last phase of the review process.^66^ This may have resulted in additional relevant studies being missed. In addition, we excluded studies on the potential prognostic value of genetic factors because they are currently difficult to include in routine ulcer assessments. Future scoping reviews should investigate this further as soon as simple genetic tests become available.

## 5 CONCLUSION

Age, gender, diabetes, smoking status, ulcer area, ulcer location, and ulcer duration at time of first assessment are potential prognostic factors that warrant a systematic review and meta‐analysis to quantify their value for predicting delayed healing of common non‐traumatic skin ulcers during routine assessment. This will contribute to optimising our understanding of ulcer healing and provide valuable information for clinical practice and guidelines.

## Supporting information


**Appendix S1.** Search strategy.

**Appendix S2.**

## References

[1] Rice JB , Desai U , Cummings AKG , Birnbaum HG , Skornicki M , Parsons N . Burden of venous leg ulcers in the United States. J Med Econ. 2014;17:347‐356. 24625244 10.3111/13696998.2014.903258

[2] Cullum N , Buckley H , Dumville J , et al. Wounds research for patient benefit: a 5‐year programme of research. Program Grants Appl Res. 2016;4:1‐304. 27583319

[3] Zhang P , Lu J , Jing Y , Tang S , Zhu D , Bi Y . Global epidemiology of diabetic foot ulceration: a systematic review and meta‐analysis†. Ann Med. 2017;49:106‐116. 27585063 10.1080/07853890.2016.1231932

[4] Dias TYAF , Costa IKF , Melo MDM , Torres SMSGSO , Maia EMC , Torres GV . Quality of life assessment of patients with and without venous ulcer. Rev Lat Am Enfermagem. 2014;22:576‐581. 25296140 10.1590/0104-1169.3304.2454 PMC4292650

[5] National Institute for Health and Care Excellence (2015) Diabetic foot problems : prevention and management. *NICE Guidline [NG19]*. 2013. Available at: https://www.nice.org.uk/guidance/ng19 26741017

[6] Leaf & Healthcare. The financial impact of pressure ulcers. Leaf Healthc. 2014;1:1‐4.

[7] National Institute for Health and Care Excellence (2015) Pressure ulcers: prevention and management. NICE Guideline [CG179]; 2014. Available at: https://www.nice.org.uk/guidance/cg179 31869019

[8] Finlayson K , Miaskowski C , Alexander K , et al. Distinct wound healing and quality‐of‐life outcomes in subgroups of patients with venous leg ulcers with different symptom cluster experiences. J Pain Symptom Manage. 2017;53:871‐879. 28063868 10.1016/j.jpainsymman.2016.12.336

[9] Ashby RL , Gabe R , Ali S , et al. VenUS IV (Venous leg Ulcer Study IV) – compression hosiery compared with compression bandaging in the treatment of venous leg ulcers: a randomised controlled trial, mixed‐treatment comparison and decision‐analytic model. Health Technol Assess. 2014;18: 1‐293. 10.3310/hta18570 PMC4781202 25242076

[10] Zimny S , Schatz H , Pfohl M . Determinants and estimation of healing times in diabetic foot ulcers. J Diabetes Complications. 2002;16:327‐332. 12200075 10.1016/s1056-8727(01)00217-3

[11] Palese A , Luisa S , Ilenia P , Laquintana D , Stinco G , di Giulio P . What is the healing time of stage II pressure ulcers? Findings from a secondary analysis. Adv Skin Wound Care. 2015;28:69‐75. 25608012 10.1097/01.ASW.0000459964.49436.ce

[12] Hippisley‐Cox J , Coupland C , Brindle P . Development and validation of QRISK3 risk prediction algorithms to estimate future risk of cardiovascular disease : prospective cohort study. BMJ. 2017;2099:1‐21. 10.1136/bmj.j2099 PMC5441081 28536104

[13] Moher D , Liberati A , Tetzlaff J , Altman DG ; The PRISMA GroupPreferred reporting items for systematic reviews and meta‐analyses: the PRISMA statement. PLoS Med. 2009;6:e1000097. 19621072 10.1371/journal.pmed.1000097 PMC2707599

[14] Kulkarni SR , Gohel MS , Wakely C , Minor J , Poskitt KR , Whyman MR . The Ulcerated Leg Severity Assessment score for prediction of venous leg ulcer healing. Br J Surg. 2007;94:189‐193. 17205494 10.1002/bjs.5597

[15] Parker CN , Finlayson KJ , Shuter P , Edwards HE . Risk factors for delayed healing in venous leg ulcers: a review of the literature. Int J Clin Pract. 2015;69:967‐977. 25831965 10.1111/ijcp.12635

[16] Edwards HE , Parker CN , Miller C , et al. Predicting delayed healing: the diagnostic accuracy of a venous leg ulcer risk assessment tool. Int Wound J. 2017;15:1‐8. 10.1111/iwj.12859. PMC7950125 29277969

[17] Centre for Reviews and Dissemination . *Systematic Reviews: CRD's Guidance for Undertaking Reviews in Health Care*. University of York: Centre for Reviews and Dissemination; 2009. 10.1016/S1473-3099(10)70065-7

[18] Levac D, Colquhoun H, O'Brien KK. Scoping studies: advancing the methodology. Implement Sci. 2010;5:69. 10.1186/1748-5908-5-69 PMC2954944 20854677

[19] Wipke‐Tevis DD , Stotts NA . Nutrition, tissue oxygenation, and healing of venous leg ulcers. J Vasc Nurs. 1998;16:48‐56. 9883147 10.1016/s1062-0303(98)90001-2

[20] Abbade LPF , Lastoria S , Rollo HDA . Venous ulcer: clinical characteristics and risk factors. Int J Dermatol. 2011;50:405‐411. 21413949 10.1111/j.1365-4632.2010.04654.x

[21] Barwell J , Ghauri ASK , Taylor M , et al. Risk factors for healing an drecurrence of chronic venous leg ulcers. Phlebology. 2000;15:49‐52.

[22] Beckert S , Witte M , Wicke C , Königsrainer A , Coerper S . A new wound‐based severity score for diabetic foot ulcers. Diabetes Care. 2006;29:988‐992. 16644625 10.2337/diacare.295988

[23] Berlowitz DR , Brandeis GH , Anderson J , Brand HK . Predictors of pressure ulcer healing among long‐term care residents. J Am Geriatr Soc. 1997;45:30‐34. 8994484 10.1111/j.1532-5415.1997.tb00974.x

[24] Cardinal M , Eisenbud DE , Armstrong DG . Wound shape geometry measurements correlate to eventual wound healing. Wound Repair Regen. 2009;17:173‐178. 19320884 10.1111/j.1524-475X.2009.00464.x

[25] Chaby G , Senet P , Ganry O , et al. Prognostic factors associated with healing of venous leg ulcers: a multicentre, prospective, cohort study. Br J Dermatol. 2013;169:1106‐1113. 23909381 10.1111/bjd.12570

[26] Christman AL , Selvin E , Margolis DJ , Lazarus GS , Garza LA . Hemoglobin A1C predicts healing rate in diabetic wounds. J Invest Dermatol. 2011;131:2121‐2127. 21697890 10.1038/jid.2011.176 PMC3174328

[27] Gohel MS , Taylor M , Earnshaw JJ , Heather BP , Poskitt KR , Whyman MR . Risk factors for delayed healing and recurrence of chronic venous leg ulcers ‐ an analysis of 1324 legs. Eur J Vasc Endovasc Surg. 2005;29:74‐77. 15570275 10.1016/j.ejvs.2004.10.002

[28] Hjerppe A , Saarinen JP , Venermo MA , Huhtala HS , Vaalasti A . Prolonged healing of venous leg ulcers: the role of venous reflux, ulcer characteristics and mobility. J Wound Care. 2010;19:474, 476, 478 passim. 21135795 10.12968/jowc.2010.19.11.79696

[29] Horn SD , Barrett RS , Fife CE , Thomson B . A predictive model for pressure ulcer outcome: the wound healing index. Adv Skin Wound Care. 2015;28:560‐572. 26562203 10.1097/01.ASW.0000473131.10948.e7

[30] Ince P , Kendrick D , Game F , Jeffcoate W . The association between baseline characteristics and the outcome of foot lesions in a UKpopulation with diabetes. Diabet Med. 2007;24:977‐981. 17559429 10.1111/j.1464-5491.2007.02189.x

[31] Jemec, GBE, Karlsmark, T, Brink‐Kjaer, T, Wulf, HC. Prediction of healing in patients with venous leg ulcers. *Acta Derm Venereol*. 1999;8:19‐23.

[32] Jones KR , Fennie K . Factors influencing pressure ulcer healing in adults over 50: an exploratory study. J Am Med Dir Assoc. 2007;8:378‐387. 17619036 10.1016/j.jamda.2007.02.011

[33] Kantor J , Margolis DJ . A multicentre study of percentage change in venous leg ulcer area as a prognostic index of healing at 24 weeks. Br J Dermatol. 2000;142:960‐964. 10809855 10.1046/j.1365-2133.2000.03478.x

[34] Kapoor A , Kader B , Cabral H , Ash AS , Berlowitz D . Using the case mix of pressure ulcer healing to evaluate nursing home performance. Am J Med Qual. 2008;23:342‐349. 18583308 10.1177/1062860608316109

[35] Labropoulos N , Wang ED , Lanier ST , Khan SU . Factors associated with poor healing and recurrence of venous ulceration. Plast Reconstr Surg. 2012;129:179‐186. 21915079 10.1097/PRS.0b013e3182362a53

[36] Margolis DJ , Allen‐Taylor L , Hoffstad O , Berlin JA . Diabetic neuropathic foot ulcers: predicting which ones will not heal. Am J Med. 2003;115:627‐631. 14656615 10.1016/j.amjmed.2003.06.006

[37] Margolis D , Berlin J , Strom B . Risk factors associated with the failure of a venous leg ulcer to heal. Arch Dermatol. 1999;135:920‐926. 10456340 10.1001/archderm.135.8.920

[38] McGinnis E , Greenwood DC , Nelson EA , Nixon J . A prospective cohort study of prognostic factors for the healing of heel pressure ulcers. Age Ageing. 2014;43:267‐271. 24366839 10.1093/ageing/aft187

[39] Meaume S , Couilliet D , Vin F . Prognostic factors for venous ulcer healing in a non‐selected population of ambulatory patients. J Wound Care. 2005;14:31‐34. 15656465 10.12968/jowc.2005.14.1.26723

[40] Moffatt CJ , Doherty DC , Smithdale R , Franks PJ . Clinical predictors of leg ulcer healing. Br J Dermatol. 2010;162:51‐58. 19785616 10.1111/j.1365-2133.2009.09397.x

[41] Monami M , Longo R , Desideri CM , Masotti G , Marchionni N , Mannucci E . The diabetic person beyond a foot ulcer: healing, recurrence, and depressive symptoms. J Am Podiatr Med Assoc. 2008;98:130‐136. 18347122 10.7547/0980130

[42] Oyibo SO , Jude EB , Tarawneh I , et al. The effects of ulcer size and site, patient's age, sex and type and duration of diabetes on the outcome of diabetic foot ulcers. Diabet Med. 2001;18:133‐138. 11251677 10.1046/j.1464-5491.2001.00422.x

[43] Park KH . A retrospective study using the Pressure Ulcer Scale for Healing (PUSH) tool to examine factors affecting stage II pressure ulcer healing in a Korean Acute Care Hospital. Ostomy Wound Manage. 2014;60:40‐51. 25211606

[44] Parker CN , Finlayson KJ , Edwards HE . Ulcer area reduction at 2 weeks predicts failure to heal by 24 weeks in the venous leg ulcers of patients living alone. J Wound Care. 2016;25:626‐634. 27827277 10.12968/jowc.2016.25.11.626

[45] Rhou YJJ , Henshaw FR , McGill MJ , Twigg SM . Congestive heart failure presence predicts delayed healing of foot ulcers in diabetes: an audit from a multidisciplinary high‐risk foot clinic. J Diabetes Complications. 2015;29:556‐562. 25804931 10.1016/j.jdiacomp.2015.02.009

[46] Ribu L , Birkeland K , Hanestad BR , Moum T , Rustoen T . A longitudinal study of patients with diabetes and foot ulcers and their health‐related quality of life: wound healing and quality‐of‐life changes. J Diabetes Complications. 2008;22:400‐407. 18413188 10.1016/j.jdiacomp.2007.06.006

[47] Scotton MF , Miot HAHA , Fernandes Abbade LP , Abbade LPF . Factors that influence healing of chronic venous leg ulcers: a retrospective cohort. An Bras Dermatol. 2014;89:414‐422. 24937814 10.1590/abd1806-4841.20142687 PMC4056698

[48] Snyder RJ , Cardinal M , Dauphinée DM , Stavosky J . A post‐hoc analysis of reduction in diabetic foot ulcer size at 4 weeks as a predictor of healing by 12 weeks. Ostomy Wound Manage. 2010;56:44‐50. 20368673

[49] Sung YH , Park KH . Factors affecting the healing of pressure ulcers in a Korean acute care hospital. J Wound Ostomy Continence Nurs. 2011;38:38‐45. 21178788 10.1097/WON.0b013e318202a67e

[50] Takahashi PY , Kiemele LJ , Chandra A , Cha SS , Targonski PV . A retrospective cohort study of factors that affect healing in long‐term care residents with chronic wounds. Ostomy Wound Manage. 2009;55:32‐37. 19174587

[51] Taylor R , Taylor A , Smyth J . Using an artificial neural network to predict healing times and risk factors for venous leg ulcers. J Wound Care. 2002;11:101‐105. 11933726 10.12968/jowc.2002.11.3.26381

[52] Vedhara K , Miles JNV , Wetherell MA , et al. Coping style and depression influence the healing of diabetic foot ulcers: observational and mechanistic evidence. Diabetologia. 2010;53:1590‐1598. 20411235 10.1007/s00125-010-1743-7

[53] Wallenstein S , Brem H . Statistical analysis of wound‐healing rates for pressure ulcers. Am J Surg. 2004;188:73‐78. 15223506 10.1016/S0002-9610(03)00294-0

[54] Wang A , Sun X , Wang W , Jiang K . A study of prognostic factors in Chinese patients with diabetic foot ulcers. Diabet Foot Ankle. 2014;5:1‐5. 10.3402/dfa.v5.22936 PMC3955769 24765244

[55] Warriner RA , Snyder RJ , Cardinal MH . Differentiating diabetic foot ulcers that are unlikely to heal by 12 weeks following achieving 50% percent area reduction at 4 weeks. Int Wound J. 2011;8:632‐637. 21951763 10.1111/j.1742-481X.2011.00860.x PMC7950518

[56] van der Wielen H , Post MWM , Lay V , Gläsche K , Scheel‐Sailer A . Hospital‐acquired pressure ulcers in spinal cord injured patients: time to occur, time until closure and risk factors. Spinal Cord. 2016;54:726‐731. 26782839 10.1038/sc.2015.239

[57] Yang GK , Cao S , Kayssi A , Dueck AD , Alavi A . Critical evaluation of delayed healing of venous leg ulcers: a retrospective analysis in Canadian patients. Am J Clin Dermatol. 2016;17:539‐544. 27480417 10.1007/s40257-016-0214-4

[58] Yotsu RR , Pham NM , Oe M , et al. Comparison of characteristics and healing course of diabetic foot ulcers by etiological classification: neuropathic, ischemic, and neuro‐ischemic type. J Diabetes Complications. 2014;28:528‐535. 24846054 10.1016/j.jdiacomp.2014.03.013

[59] Zimny S , Pfohl M . Healing times and prediction of wound healing in neuropathic diabetic foot ulcers: a prospective study. Exp Clin Endocrinol Diabetes. 2005;113:90‐93. 15772900 10.1055/s-2004-830537

[60] Thompson SG , Higgins JPT . How should meta‐regression analyses be undertaken and interpreted? Stat Med. 2002;21:1559‐1573. 12111920 10.1002/sim.1187

[61] Riley RD , Lambert PC , Abo‐Zaid G . Meta‐analysis of individual participant data: rationale, conduct, and reporting. BMJ. 2010;340:521‐525. 10.1136/bmj.c221 20139215

[62] Hayden JA , Van Der Windt DA , Cartwright JL , Côté P . Assessing bias in studies of prognostic factors. Ann Intern Med. 2013;144:427‐437. 10.7326/0003-4819-158-4-201302190-00009 23420236

[63] Westby MJ , Dumville JC , Stubbs N , et al. Protease activity as a prognostic factor for wound healing in venous leg ulcers. Cochrane Database Syst Rev. 2018;2018:1‐87. 10.1002/14651858.CD012841.pub2 PMC6513613 30171767

[64] Jenkins D , Bujkiewicz S , Martina R , Dequen P , Abrams KR . Methods for the inclusion of real world evidence in network meta‐analysis. *arXiv Prepr*.; 2018. 10.1186/s12874-021-01399-3 PMC8502389 34627166

[65] Tricco AC , Lillie E , Zarin W , et al. A scoping review on the conduct and reporting of scoping reviews. BMC Med Res Methodol. 2016;16:1‐10. 26857112 10.1186/s12874-016-0116-4 PMC4746911

[66] Arksey H , O'Malley L . Scoping studies: towards a methodological framework. Int J Soc Res Methodol. 2005;8:19‐32.

