# Supplementary material for: Potential prognostic factors for delayed healing of common, non‐traumatic skin ulcers: A scoping review
Source: Int Wound J. 2019 Feb 28;16(3):800–12. doi: 10.1111/iwj.13100 (PMC6563199; doi:10.1111/iwj.13100)
Supplement: Supplementary file 1 — Appendix S1. Search strategy. [file IWJ-16-800-s001.docx]

**Appendix 1 –Search strategy**

| 1 | Validat*.mp |
| --- | --- |
| 2 | Predict*.ti. |
| 3 | Rule*.mp. |
| 4 | 1 or 2 or 3 |
| 5 | Predict*.mp. |
| 6 | (Outcome* or Risk* or Model*).mp. [mp=title, abstract, original title, name of substance word, subject heading word, keyword heading word, protocol supplementary concept word, rare disease supplementary concept word, unique identifier, synonyms] |
| 7 | 5 and 6 |
| 8 | (History or Variable* or Criteria or Scor* or Characteristic* or Finding* or Factor*).mp. [mp=title, abstract, original title, name of substance word, subject heading word, keyword heading word, protocol supplementary concept word, rare disease supplementary concept word, unique identifier, synonyms] |
| 9 | (Predict* or Model* or Decision* or Identif* or Prognos*).mp. [mp=title, abstract, original title, name of substance word, subject heading word, keyword heading word, protocol supplementary concept word, rare disease supplementary concept word, unique identifier, synonyms] |
| 10 | 8 and 9 |
| 11 | Decision*.mp. |
| 12 | (Model* or Clinical* or Logistic Models).mp. [mp=title, abstract, original title, name of substance word, subject heading word, keyword heading word, protocol supplementary concept word, rare disease supplementary concept word, unique identifier, synonyms] |
| 13 | 11 and 12 |
| 14 | Prognostic.mp. |
| 15 | (History or Variable* or Criteria or Scor* or Characteristic* or Finding* or Factor* or Model*).mp. [mp=title, abstract, original title, name of substance word, subject heading word, keyword heading word, protocol supplementary concept word, rare disease supplementary concept word, unique identifier, synonyms] |
| 16 | 14 and 15 |
| 17 | 4 or 7 or 10 or 13 or 16 |
| 18 | exp Diabetic Foot/ |
| 19 | exp Foot Ulcer/ |
| 20 | (diabet* adj3 ulcer*).tw. |
| 21 | (diabet* adj3 (foot or feet)).tw. |
| 22 | (diabet* adj3 wound*).tw. |
| 23 | (diabet* adj3 defect*).tw. |
| 24 | 18 or 19 or 20 or 21 or 22 or 23 |
| 25 | (varicose ulcer* or venous ulcer* or leg ulcer* or stasis ulcer* or crural ulcer* or ulcus cruris or ulcer cruris or foot ulcer*).tw. |
| 26 | exp Leg Ulcer/ |
| 27 | 25 or 26 |
| 28 | exp Pressure Ulcer/ |
| 29 | (pressure adj (ulcer* or sore*)).tw. |
| 30 | (decubitus adj (ulcer* or sore*)).tw. |
| 31 | (bedsore* or (bed adj sore*)).tw. |
| 32 | 28 or 29 or 30 or 31 |
| 33 | 24 or 27 or 32 |
| 34 | 17 and 33 |
| 35 | limit 34 to (english language and humans) |
