# Supplementary material for: Potential prognostic factors for delayed healing of common, non‐traumatic skin ulcers: A scoping review
Source: Int Wound J. 2019 Feb 28;16(3):800–12. doi: 10.1111/iwj.13100 (PMC6563199; doi:10.1111/iwj.13100)
Supplement: Supplementary file 2 — Appendix S2. [file IWJ-16-800-s002.docx]

Appendix 2

| **Author** | **Potential factors as labelled in each study** |
| --- | --- |
| ***Diabetic foot ulcers****(15 studies)* | |
| Beckert | Multiple ulcers, probing to the bone, site of ulcer and nonpalatable pulse |
| Christman | age, sex, ethnicity, smoking, peripheral artery disease, SBP,DBP, pulse, temp, neuropathy, BMI, wound number, white blood cell count, HbA1c,triglycerider |
| Ince | ulcer site, ulcer area, depth, time between ulcer onset and clinic visit, sex, Age (<50 and every 10 years), years since diabetes diagnosis, diabetes type, IMD quartile, sepsis, arteriopathy, denervation |
| Margolis | Sex, age,curative center ulcer grade, ulcer duration of oldest wound, ulcer area of larest wound, no of wounds |
| Monami | Greenfield index of disease severity, Smoking, geriatric depression scale, Ankle-brachial index, ulcer duration, Ulcer area at baseline, University of Texas score, HbA1c |
| Oyibo | age, sex, diabtetes type, diabetes duration, ischaemia, ulcer size, ulcer site, ulcer depth, infection |
| Rhou | Age, sex, diabetes duration, smoking status, current alcohol consumption, peripheral vascular disease, chronic kidney disease, congestive cardiac failure. Cardiovascular agent use, beta blocker use, calcium channel blocker use, frusemide use, statin use, antibiotic use duration, ulcer area, ulcer depth, ulcer infection, lab characteristics (eGFR, HbA1c, Alumin, Bilirubin, ALP, GGT, ALT, AST, uric acid) |
| Ribu | Physical functioning, role limitation-physical, bodily pain, general health, vitality, social functioning, role limitation-emotional, mental health, physical summary score and mental summary score |
| Snyder | Ulcer area at baseline, % area reduction at 4 weeks |
| Vedhara | age, sex, martial status, income, diabetes type, BMI, neuropathy, ischaemia, smoking, chronic co-morbid conditions (arthritis, asthma, angina, hypertension, cancer, heart disease), depression, anxiety and coping, ulcer infection, no of previous ulcers, area, HbA1c |
| Wang | sex, age, smoker, education, diabetes duration, family diabetes history, electrocardiogram, retinopathy, neropathy, SBP, DBP, ulcer duration, ulcer history, wagner grade, area, site of ulcer, fasting glucose, HBA1c, Urine Alb, BUN, Cr, TP, ALB, TG, TCH, WBC, N, Hb and ESR,ABI, TcPO2, gram-positive bacteria |
| Warriner | 90% area reduction at 4,6 and 8 weeks |
| Yotsu | Age, sex, diabetes follow up, retinopathy, renal dialysis, ABI, SPP, tcpO2, Hemoglibin, Serum albumin, eGFR, ABI, SPP, tcpO2, Hemoglibin, Serum albumin, eGFR |
| Zimney | Wound area and radius of wound |
| Zimney | Wound area, area reduction, wound radius and wound radius reduction |
| ***Pressure ulcers (10 studies)*** | |
| Berlowitz | age >=75, sex, residing in nursing home, recent admission, incontinent, immobile, never transferred from bed, dependent in eating, coma, multiple sclerosis, quadriplegia, hemiplegia, terminal ilness, dehydration, renal dialysis, oxygen therapy UTI, not recieving rehabilitation sevices, statis ulcer |
| Horn | wound duration, history of wounds, stage, days from first to last encounter, worst braden score, |
| Jones | Age, sex, marital status, race, insurance, primary care giver (eg, family, self and home health), congestive HF, CVD, hypertension, DVT, diabetes, cancer, arthritis, neuropathy, dementia, MS/PD, CVA, Blood disorder, pulmonary disease, GI disorder, depression, renal disorder, thyroid disorder, electrolyte imbalance, osteomyelitis, skin disorder, sensory disorder, total comorbids, obesity, smoking, malnutrition, ulcer size (5cm groups and overall), depth, severity score, location, stage, exudate amount, exudate type, necrosis type, more than one wound, no of secondary ulcers, any secondary treatment, dressing type changed, growth factor applied, silver based product applied, topical antiseptic applied, toxic cleanse applied, bedridement performed, mechanical debridment performed, sharp debridement preformed, enzymatic debridement performed, autolytic debridement performed, |
| Kapoor | age <65, sex, intact bed mobility, intact transfer from bed, incontinence (bowel and urinary), absense of paraplegia and quadriplegia, absense of end-stage illness, stage 2 ulcer, stage 3 ulcer,history of resolved ulcer |
| McGinnis | age, gender, ethnicity, speciality, co-morbidity, nutritional status, smoking, medication, pain,neuropathy, arterial disease, ulcer severity, size, duration and tissue type and surrounding skin condition.individual Braden risk scale factors |
| Park | sex, age<65, major diseases (neurovascular, cardiovascular, respiritory, digestive, musculoskeletal, cancer and other), diabetes, hypertension, peripheral arterial disease,incontinence, stool form, smoking,application of restraints, chemo, antidepressants, vitamins, mean arterial pressure, wound location, infection, size, exudate amount, initial tissue type, dressing method, MUST score, Braden score, history of ulcers, serum hemoglobin, serum albumin |
| Sung | sex, age<65, major diseases (neurovascular, cardiovascular, respiritory, digestive, musculoskeletal, cancer and other), diabetes, hypertension, peripheral arterial disease,incontinence, stool form, smoking,application of restraints, chemo, antidepressants, vitamins, mean arterial pressure, serum hemoglobin, serum albumin, wound location, infection, size, exudate amount, initial tissue type, dressing method, MUST score, Braden score, history of ulcers |
| Takahashi |  |
| Wallenstin | Wound area |
| Wielen | sex, age (18-35, 36-50, 51-65, >66),reason for admission, time since lesion, aetiology, lesion level, AIS injury severity, PU at admission, localisation, grade of first admission |
| ***Venous leg ulcers*** *(17 studies)* | |
| Abbade | Age (>50,>60, >70), sex, BMI(>25,>30,>35,>40), , severe lipodermatosclerosis, superficial thrombophlebitis history, DVT history, diabetes, aterial hypertension, multiparity, isolated SVSI, isolated DVSI, GS severe reflux, SVSI+DVSI, DVSI + incompetence of perforators, SVSI+ incompetence of perferators, SVSI+DVSI+incompetence of perforators, large ulcer area, previous history of ulcer |
| Barwell | age, sex, mobility score, rheumatoid arthritis, diabetes, , superficial venous reflux + surgery, superficial venous reflux + no surgery, deep venous reflux, mixed venous reflux, popliteal vein reflux, ulcer size, ulcer chronicity |
| Chaby | age, sex, bmi, comorbidity (cardiovascular risk factors, cardiac insufficience, rheumatological disease, orthopaedic surgery, prior DVT, renal insufficiency, prior venous surgery), wound area, duration, recurrent wond, granulation tissue %, VAS score, venous duplex ultrasonography (iscolated, DVI, popliteal vein reflux), lipodermatsclerosis, white atrophy, ankle joint ankylosis, superficial vein surgery, home owner, rented home, free universal healthcare, long term illness, private health insurance, living alone, urban/rural, income, education level, marital status, employment status, depression, coppersmith self-esteem score, revens coloured progressive matrices, mini mental state examination, area reduction at 4wks, adherence to compression, albuminaemia, anemia, ADL score, ADL score<3 |
| Cardinal | sex, height, weight, BMI, age, ABPI, Anemia, diabetes, stroke, hypertension, PVD, congestive heart failure, Rheumatoid arthritis, osteoarthritis, alcohol consumption, smoking, DVT, hyperpigmentation, varicosities, lipermatosclerosis, dermatitis, leg mobility, ulcer position, shape change, initial wound area, wound duration, swelling, initial shape, reccuring ulcer, bilateral ulcer,wound exudate, necrotic tissue, pain |
| Gohel | age, sex, history of DVT, rheumatoid arthritis, segmental deep reflux, total deep reflux, incompetent calf veins, superficial venous reflux - no surgery, superficial venous reflux - with surgery, location, ulcer chronicity |
| Hjerppe | age, sex, smoking, BMI, diabetes, venous clinical scoring system, over 5 medications, common femoral, popliteal, posterior tibial, saphenous magna, saphenous parva, saphenous magna residual, walker stick and no utility in use |
| Jamec | Age, wound duration, wound size and deep vein involvment, haemoglobin, white blood cell count, sodium, potassium, creatinine, glucose, albumin, ASAT, alkaline phosphatase |
| Kantor | Area reduction at 2,3 and 4 weeks and change between weeks 1-2 and 3-4 |
| Labropoulos | BMI>25, DVT (no, single and multi), age, area |
| Margolis | Age, ankle brachial index, venous flow index, public assistance or self-pay, sex, non white, , thyroid disease, diabetes, DVT, hypertension, MI, cerebral vascular accident, angina, hip or knee replacemen surgery, unable to walk 1 block,varicose veins present, dermatitis, lipodermatosclerosis, ankle brachial index <0.8, no limb edema, Area, wound duration, limb ulcer number,venous ligation or stripping, wound margin undetermined, >50% wound covered with eschar, >50% wound covered with fibrin, wound devrided surgically |
| Meaume | sex, age >80, BMI >30, hypertension, dyslipidaemia, diabetes, lower limb arterial disease, history of orphopedic surgery, history of venous surgery, history of DVT, smoking, ulcer duration >3 months, largest length >10cm, more than one current ulcer, recurrent ulcer and atleast one sign of infection |
| Moffett | Age (<65,65-74,75-84,85+), sex, mobility (walks freely, walks outdoors with aid, walks with aid indoor and bed bound), ankle function, equnus deformity, medical history (diabetes, DVT, thrombophlebitis, lipodermatosclerosis, atrophie blanche, varicose eczema), presence of reduced venous refilling, prolonged popliteal reflux, presence of deep venous incompetence, ulcer size >10cm, ulcer duration, ulcer aetiology, compression, bacteriology (clinical cellulits, stapahlyloccus aureus, haemolytic streptococcus, anerobes, pseudomonas, number of species), |
| Parker | rheumatoid arthritis, gout, autoimmune disease, DVT in study leg, oedema, fixed ankle joint, walking aid, aching in study leg, allopurinol, salbutamol, antidepressants, , calf size reduced >2cm at 2 week, , lives alone and GDS, haemosiderosis, lymphoedema, treated with compression, 25% area reductin at 2 weeks, exudate, tissue type, PUSH score, physical component sumary scale, ulcer duration, ulcer area, pain |
| Scotton | sex, age, post thrombotic etiology, hypertension, diabetes, compliance with rest, duration of topical antibiotic use, theraputic use of systemic antibiotics, duration of ulcer, ulcer location, area, critical colonizatio, infection,compliance with dressing, compliance with compression, duration of debriding |
| Taylor | Age, Sex (male), Lives alone (yes), Smoker (current or ex), Previous leg ulcer (yes), Diabetes (yes), Rheumatoid arthritis (yes), Treatment for varicose veins (yes), Treatment for DVT (yes), Mobility (full), Peripheral vascular disease (yes), Body mass index (kg/m2), Systolic blood pressure (mmHg), Diastolic blood pressure (mmHg), Ankle brachial pressure index (left), Ankle brachial pressure index (right), Treatment given (4LHCB), Treatment given (3LHCB), Ulcer status (new),Time since first ulceration (months), Age of main ulcer (weeks), Ulcer position (medial malleolus, malleolus, anterior gaiter, gaiter, lateral gaiter,medial gaiter), Study leg (left), Level of pain experienced (none, mild, moderate, severe), Exudate condition, Condition of surrounding skin (inflammation), Condition of surrounding skin (indurated), Condition of surrounding skin (oedema), Condition of surrounding skin (dry/flaky), Wound base (necrotic), Wound base (sloughy), Wound base (granulating), Wound base (epithelialising), area |
| Wipke-Tevis | Albumin, vitamin C, zinc, reginal perfusion index, TLC, tissue oxygenation, vitamin A |
| Yang | age, age>65, sex, comorbidities (hypertension, dyslipidemia, smoking, diabetes, history of DVT, trauma leadingto ulcer, osteoarthritis, obesity, coronary artery disease, peripheral arterial disease), vascular surgery, largest ulcer size |
